# The need for non-technical skills education in orthopedic surgery

**DOI:** 10.1186/s12909-023-04196-2

**Published:** 2023-04-19

**Authors:** Khalid H. Alzahrani, Raid A. Abutalib, Ahmed M. Elsheikh, Laura K. Alzahrani, Khalid I. Khoshhal

**Affiliations:** 1 Department of Orthopedic Surgery, Security Forces Hospital, PO Box 14799, 21955 Makkah, KSA Saudi Arabia; 2 Division of Orthopedics, Department of Surgery, Prince Mohammed bin Abdul-Aziz National Guard Hospital, Almadinah Almunawwarah, Medina, PO Box 3684, Saudi Arabia; 3 Department of Quality and Patient Safety, Security Forces Hospital, PO Box 14799, 21955 Makkah, KSA Saudi Arabia; 4 College of Medicine, Fakeeh College of Medical Sciences, Jeddah, 23323 KSA Saudi Arabia

**Keywords:** Communication, Non-technical skills, Operating room, Orthopedic Surgeons, Patient care, Patient safety

## Abstract

**Background:**

The issue of surgical safety has increased significantly over the last few decades. Several studies have established that it is linked to non-technical performance, rather than clinical competencies. Non-technical skills can be blended with technical training in the surgical profession to improve surgeons’ abilities and enhance patient care and procedural skills. The main goal of this study was to determine orthopedic surgeons’ requirements of non-technical skills, and to identify the most pressing issues.

**Methods:**

We conducted a self-administered online questionnaire survey in this cross-sectional study. The questionnaire was piloted, validated, pretested, and clearly stated the study’s purpose. After the pilot, minor wording and questions were clarified before starting the data collection. Orthopedic surgeons from the Middle East and Northern Africa were invited. The questionnaire was based on a five-point Likert scale, the data were analyzed categorically, and variables were summarized as descriptive statistics.

**Results:**

Of the 1713 orthopedic surgeons invited, 60% completed the survey (1033 out of 1713). The majority demonstrated a high likelihood of participating in such activities in the future (80.5%). More than half (53%) of them preferred non-technical skills courses to be part of major orthopedic conferences, rather than independent courses. Most (65%) chose them to be face-to-face. Although 97.2% agreed on the importance of these courses, only 27% had attended similar courses in the last three years. Patient safety, infection prevention and control, and communication skills were ranked at the top as topics to be addressed. Moreover, participants indicated they would most likely attend courses on infection prevention and control, patient safety and teamwork, and team management.

**Conclusion:**

The results highlight the need for non-technical skills training in the region and the general preferences regarding modality and setting. These findings support the high demand from orthopedic surgeons’ perspective to develop an educational program on non-technical skills.

**Supplementary Information:**

The online version contains supplementary material available at 10.1186/s12909-023-04196-2.

## Background

It has been demonstrated that there is an increasing need for non-technical training to improve patient care and safety from an institutional perspective, in the Middle East and North African regions. Consequently, there is growing global interest in educating all healthcare providers on non-technical skills [1]. In addition, over the past three decades, there has been an increasing interest in linking the non-technical skills of healthcare providers with patient safety [2–4]. Some studies have demonstrated the rising demand for this skill set from surgeons’’ perspectives [5]. Others have studied how a lack of non-technical skills affects patient safety and causes harm, believing that it negatively affects the technical performance of medical professionals [6]. Moreover, it has been reported that several adverse events are mainly due to a failure in non-technical performance, rather than clinical proficiency [7, 8].

The demand for these skills has been largely identified through the rise of adverse events reported in surgical specialties, highlighting the value of non-technical skills, such as communication, clinical leadership, shared decision-making, and conflict management, on patient safety [9, 10]. Flin defined these skills as “the cognitive, social, and personal resource skills that complement technical skills and contribute to safe and efficient task performance” [11, 12]. These skills can be acquired and improved through practice, reflection, feedback, and repetition [12]. Multiple aspects of complex patient care require a particular type of non-technical skill, for example, communication, teamwork, breaking bad news, professionalism, and leadership. In complex clinical settings, such as operating theaters or emergency settings, inadequate non-technical abilities have been linked to undesirable impacts on patient safety [13–17]. For instance, surgeons are vulnerable to various stresses that can negatively impact patient outcomes; in such situations, studies have shown that non-technical abilities, such as effective communication, can help minimize stress [15, 18]. Therefore, healthcare providers’ non-technical skills competencies significantly enhance patient care outcomes.

Currently, some efforts are being made to address the demand for non-technical skills training using educational interventions, in the form of stand-alone courses or integrated within clinical programs in undergraduate or postgraduate training [2, 3]. Still, several important aspects, such as decision-making, leadership, communication skills, and teamwork, are overlooked. This lack of non-technical skills training generates a considerable knowledge gap between the academic and clinical sides, when graduates start caring for their patients. Therefore, some efforts are being implemented to impart this type of education, by including non-technical skills training in the curricula of postgraduate programs. For instance, Canadian residency training programs have implemented the CanMEDS framework, incorporating six non-technical competencies, to graduate medical experts [19, 20]. Moreover, the Accreditation Council for Graduate Medical Education (ACGME) has introduced six core competencies to improve teaching and learning for medical graduates [21]. Similar efforts are being implemented to introduce essential skills to undergraduate students, such as Saudi Med and GMC, to promote excellence standards and enrich graduate students’ patient care abilities [22, 23]. Additionally, there are stand alone programs like “Team Strategies and Tools to Enhance Performance and Patient Safety” (TeamSTEPPS), which provide structured communication training to improve patient safety [24]. Moreover, there are rising structured courses designed especially for surgeons, such as Non-Technical Skills for Surgeons (NOTSS), to build up taxonomy and training systems to improve the safety for surgical patients [25].

However, in orthopedic specialties, non-technical skills have not yet been established as a fundamental component of residency training, or as essential supplementary programs linked to major orthopedic scientific activities. The challenge is to make this type of education more relevant and attractive to orthopedic surgeons. Furthermore, the types of required training and their relevance, based on surgeons’ experience and demands, have not been sufficiently explored or defined for orthopedic specialties. As most current non-technical skills courses are general and not specialty-based, many surgeons may consider them irrelevant. The primary purpose of this study was to explore orthopedic surgeons’ needs for non-technical skills against this background, and identify the most relevant topics that need to be addressed.

## Materials and methods

### Design

A cross-sectional questionnaire-based survey was used to evaluate the demands for non-technical skills among orthopedic surgeons in the Middle East and North Africa (MENA). We contacted the AO orthopedic association chairs in MENA region to obtain a list of all practicing members in their respective countries. The self-administered questionnaire, developed in English based on published guidelines [26–29], sought to identify orthopedic surgeons’ perceived needs for non-technical training. The questionnaire covered ten items and was made securely available online, to explore participants’ preferred topics. Each topic was rated on a five-point Likert-type scale. Additionally, we aimed to determine respondents’ likelihood of participating in complementary non-technical skills educational activities, when these were combined with major orthopedic scientific activities. We also aimed to compare our findings against participants’ demographic data, such as years of experience, gender, and workplace.

The survey was pretested to determine face and content validity, by asking five orthopedic surgeons and one consultant in medical education to go through the questionnaire, and offer suggestions and feedback. Subsequently, their areas of concern were modified. Furthermore, we piloted the questionnaire for five other orthopedic surgeons, to ensure transparency and determine the approximate time required for completing it. The collection started after a short pilot phase, which resulted in minor changes in wording and clarification of questions, before we began the actual data collection.

### Procedure

After obtaining approval from the Medical Research Center of the Security Forces Hospital (IRB protocol approval number 0380–03092) and a list of orthopedic surgeons from orthopedic associations in the MENA region, the surgeons were contacted electronically via direct invitations between March and April 2022. The purpose of the study was clearly described in a cover letter. An individual’s decision to complete the questionnaire was considered an indication of their consent to participate. Once the responses were received, we removed all identifying information, and replaced it with a number code to ensure confidentiality.

### Data analysis

Raw data were collected, converted using a Microsoft Excel® spreadsheet, and analyzed using SPSS version 20 (IBM Corp., Armonk, NY, USA). Mean values and standard deviations (SD) were used to describe continuous data, whereas absolute and relative frequencies were used to represent categorical variables. The data were analyzed on a five-point Likert scale, and variables were summarized as descriptive statistics (i.e., frequency, percentage, mean, median, mode, and standard deviation).

## Results

### Demographic data

The questionnaire was sent to 1,713 orthopedic surgeons across the Middle East and North Africa (MENA) region using the SurveyMonkey® platform. Of these, 1033 (60%) completed the survey, and all responses were included.

The participants represented different orthopedic subspecialties; the highest representation was from orthopedic trauma (40.3%). Most participants (53.9%) had been practicing orthopedic surgery for more than ten years (Table 1). The participants represented all countries listed in the original invitation, with the highest three being Saudi Arabia (26.5%), Egypt (20.2%), and Kuwait (8.6%) (Table 2). Only 27 female orthopedic surgeons were enrolled in the study, while the male surgeons represented 97.4% of the participants (Table 3). The majority of the participants (73%) held board certificates or its equivalents in orthopedic surgery (Table 3). More than 57% were working in government hospitals (Table 4).

**Table 1:** Participant distribution by sub-specialty and years of practice

Specialty	Years of practice					Total	%
	< 5	6–10	11–15	> 15 years	Not Defined		
Orthopedic trauma	92	136	84	104	0	416	**40.3**
General orthopedic surgery	68	35	25	46	1	175	**16.9**
Arthroplasty and adult reconstruction	9	30	42	47	0	128	**12.4**
Sports medicine	5	30	23	46	1	105	**10.2**
Pediatric orthopedic Surgery	5	19	17	26	0	67	**6.5**
Hand surgery		9	8	18	0	35	**3.4**
Spine surgery	2	8	11	13	0	34	**3.3**
Upper extremity surgery	1	8	10	10	0	29	**2.8**
Ilizarov/deformity correction		5	6	5	0	16	**1.6**
Others	11	2	5	10	0	28	**2.7**
Total	**193**	**282**	**231**	**325**	**2**	**1033**	**100.00**

**Table 2:** Participant distribution by country

Country of practice	n	%
Saudi Arabia	274	26.5
Egypt	209	20.2
Kuwait	89	8.6
Jordan	76	7.4
Iraq	67	6.5
Pakistan	66	6.4
Oman	51	4.9
Sudan	50	4.8
UAE	31	3.0
Iran	22	2.1
Bahrain	22	2.1
Tunisia	15	1.5
Lebanon	11	1.1
Morocco	5	0.5
Others	45	4.4
Total	**1033**	**100.0**

**Table 3:** Participant distribution by gender and certification

	Board Certified		Non-board certified		Total number	Total percentage
Gender	n	%	n	%		
**Women**	14	1.4%	13	1.3%	27	2.6
**Men**	744	72.0%	262	25.4%	1006	97.4
**Total**	758	73.4%	275	26.6%	1033	100.0

**Table 4:** Participant distribution according to the type of working institutes

Organization	n	%
Governmental hospital	595	57.7
Academic (University hospital)	208	20.2
Private hospital	199	19.3
Healthcare organization	17	1.5
Others	14	1.4
Total	**1033**	**100.0**

### Course preferences

More than half (53%) the participants preferred that the non-technical skills course be part of a major orthopedic event, rather than an independent course. Additionally, most participants (65%) preferred to attend face-to-face courses compared to virtual ones (Table 5). Interestingly, 27% had participated in courses on non-technical skills in the last three years, with no significant difference detected using Chi-square in this finding when compared across the country of practice (p = 0.740) and years of experience (p = 0.68), where Cl is 95%.

**Table 5:** Preferred mode of course delivery and method of participation

Method of course delivery	n	%	Method of Participation	n	%
Independent course	486	47.0	Face-to-face	668	64.7
Part of major orthopedic activity	547	53.0	Virtual	365	35.3
Total	**1033**	**100.0**	**Total**	**1033**	**100.0**

**Fig. 1:**
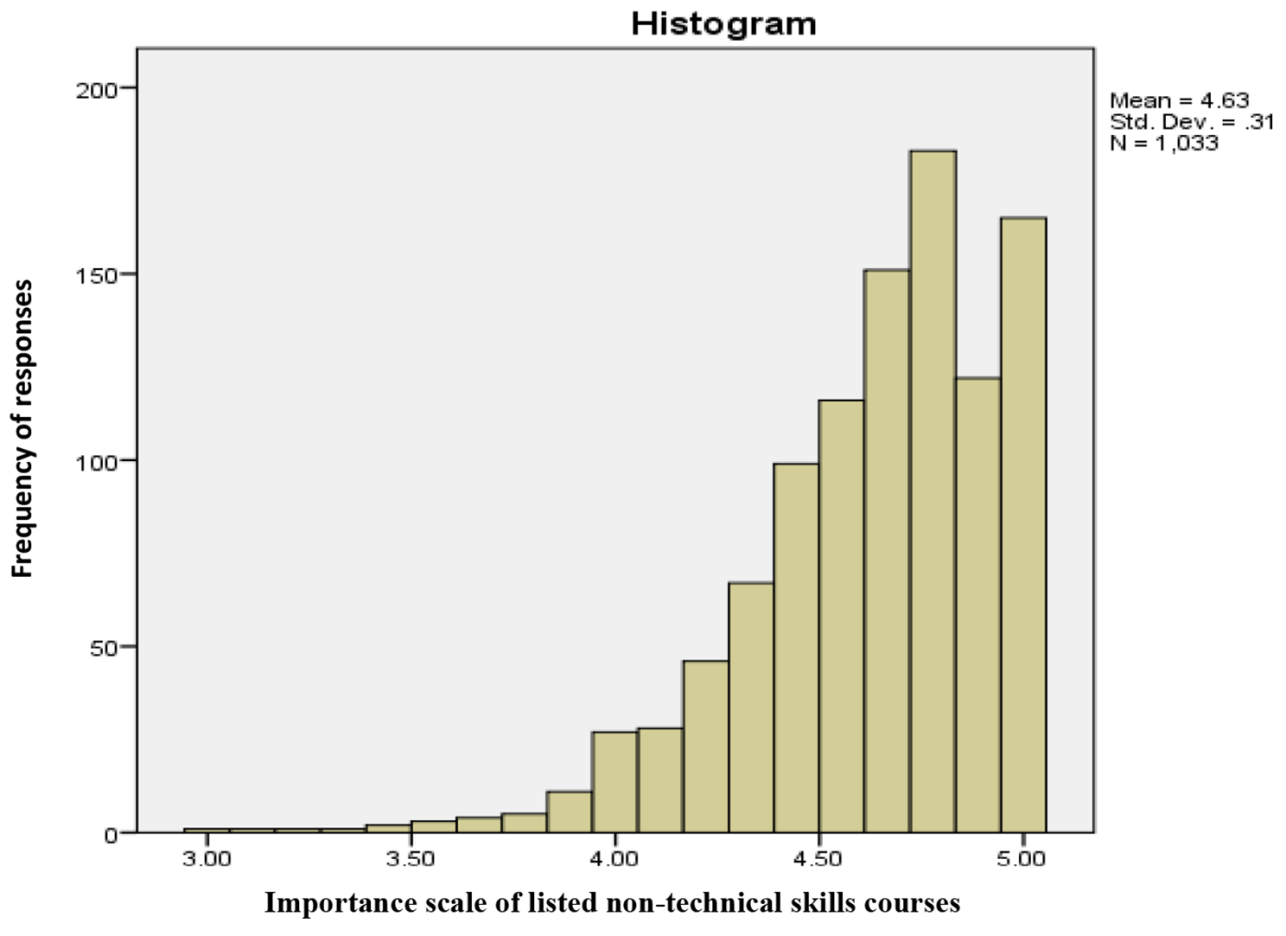
Participant responses to the importance of the listed non-technical skills courses

**Fig. 2:**
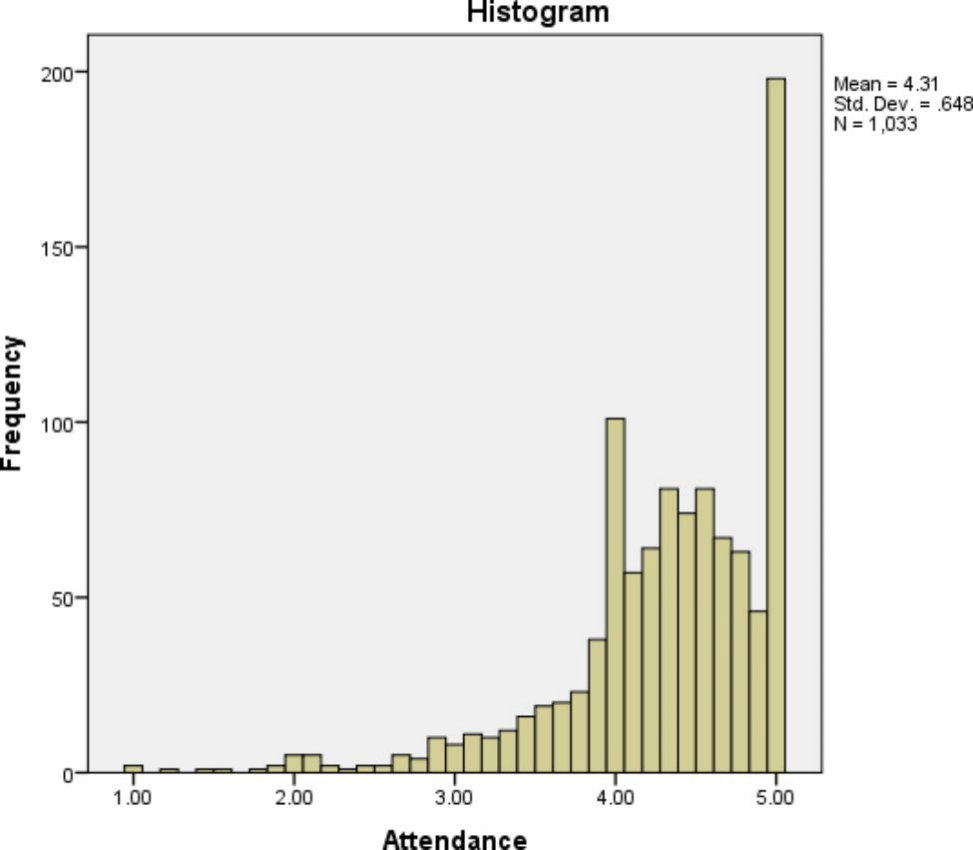
Participant responses on the desire to attend one of the listed non-technical skills courses

Most participants agreed that listed non-technical skills were essential to their practice (mean: 4.63; 97.2% scored ≥ 4 on weighted score) (Fig. 1). Additionally, the majority demonstrated a high likelihood of participating in a technical skills course in the future (mean: 4.31; 80.5% scored ≥ 4 on the weighted score) (Fig. 2). Moreover, there was no statistically significant difference related to an agreement regarding the listed courses’ importance and the desire to attend one, when compared by gender (p = 0.075), years of experience (p = 0.195), or country of practice (p = 0.557).

Of all topics, patient safety, infection prevention and control, and communication skills emerged as the top-ranked priorities to be addressed (Table 6). Further, the participants indicated that they would most likely attend courses related to infection prevention and control, patient safety and teamwork, and team management (Table 7).

**Table 6:** Weighted score of the listed topics for the participants

Importance	Useless (1)		Not important (2)		Not sure (3)		Important (4)		Very important (5)		Weighted score team	Weighted score %
	Count	Row n %	Count	Row N %	Count	Row N %	Count	Row N %	Count	Row N %		
Patient safety	0	0.0	1	0.1	5	0.5	119	11.5	908	87.9	5033	97.4
Infection prevention and control	0	0.0	0	0.0	12	1.2	140	13.6	881	85.3	5001	96.8
Communication skills	0	0.0	2	0.2	10	1.0	208	20.1	813	78.7	4931	95.5
Teamwork and team management	0	0.0	6	0.6	13	1.3	221	21.4	793	76.8	4900	94.9
Doctor-patient relationship	0	0.0	4	0.4	44	4.3	237	22.9	748	72.4	4828	93.5
Professionalism	0	0.0	1	0.1	28	2.7	294	28.5	710	68.7	4812	93.2
Clinical leadership skills	1	0.1	8	0.8	24	2.3	324	31.4	676	65.4	4765	92.3
Medicolegal issues	0	0.0	5	0.5	65	6.3	317	30.7	646	62.5	4703	91.1
Patient-doctor shared decision making	1	0.1	6	0.6	71	6.9	408	39.5	547	53.0	4593	88.9
Telehealth and virtual patient care	9	0.9	51	4.9	253	24.5	395	38.2	325	31.5	4075	78.9

Weighted score percentage = (relative important scores / (number of respondents*5))*100.

Weighted score = (((count of useless responses for topic X * (1)) + (count of not important responses for topic X * (2)) + (count of not sure responses for topic X * (3)) + (count of important responses for topic X * (4)) + (count of very important responses for topic X * (5))) * (total number of responses*5) )/100.

**Table 7:** Relative likelihood of attending a course on one of the listed topics

Topic attendance	Very unlikely (1)		Unlikely (2)			Not sure (3)			Likely (4)		Very likely (5)		Weighted score score	Weighted score %
	Count	Row N %	Count	Row N %	Count		Row N %	Count		Row N %	Count	Row N %		
Infection prevention and control	12	1.2	25	2.4	69		6.7	286		27.7	641	62.1	4618	89.4
Patient safety	10	1.0	22	2.1	65		6.3	342		33.1	594	57.5	4587	88.8
Teamwork and team management	12	1.2	30	2.9	70		6.8	369		35.7	552	53.4	4518	87.5
Clinical leadership skills	8	0.8	34	3.3	73		7.1	374		36.2	544	52.7	4511	87.3
Communication skills	13	1.3	48	4.6	83		8.0	367		35.5	522	50.5	4436	85.9
Professionalism	15	1.5	34	3.3	88		8.5	396		38.3	500	48.4	4431	85.8
Medicolegal issues	8	0.8	27	2.6	120		11.6	397		38.4	481	46.6	4415	85.5
Doctor-patient relationship	15	1.5	46	4.5	141		13.6	414		40.1	417	40.4	4271	82.7
Patient-doctor shared decision making	13	1.3	36	3.5	155		15.0	436		42.2	393	38.0	4259	82.5
Telehealth and virtual patient care	29	2.8	67	6.5	185		17.9	386		37.4	366	35.4	4092	79.2

Weighted score percentage = (weighted score scores / (number of respondets*5))*100

= (((count of very unlikely responses for topic X * (1)) + (count of unlikely responses for topic X * (2)) + (count of not sure responses for topic X * (3)) + (count of likely responses for topic X * (4)) + (count of very likely responses for topic X * (5))) * (total number of responses*5) )/100

## Discussion

This study was based on a pilot study conducted in 2019 ^5^. Compared to the pilot, the current study included over nine times (1033) more orthopedic surgeons from the MENA region. The participants represented different orthopedic sub-specialties. Although trauma surgeons were the highest in number (40.3%), other specialties were fairly represented. More than half of the respondents (53.9%) had more than 10 years of experience, and 73% were certified orthopedic surgeons, which gives more credibility to their responses.

This study is aligned with the growing global demand for teaching healthcare providers non-technical skills to improve patient care [5, 10, 30]. The results showed that although only 27% of the participants had attended non-technical courses in the last three years, most of them considered these to be essential. Most of them were willing to receive formal education on all 10 survey topics, and felt that these courses were crucial for their practice. The choice of topics in the survey was based on a pilot study, and a thorough literature review of the most relevant subjects in this field. However, the fact that only 27% of the participants had attended non-technical skills courses, although the vast majority were motivated to participate, indicates that there are not enough courses offered in the region, for this important field. The reasons for this could be gleaned from Scot et al.’s systematic review, which identified three critical forces that guide the development and application of non-technical skills in low and middle-income countries: overburdened healthcare systems, lack of institutional empowerment, and deficiencies in provider training [30].

Patient safety, infection prevention and control, communication skills, teamwork, and team management were considered the highest priority to be addressed, and were also the topics with the highest likelihood of attendance. However, in the pilot study, professionalism, patient safety, and medicolegal issues had emerged as topics with the highest priority [5]. In the present study, patient safety remains in the top three; however, infection prevention and control are now gaining importance, possibly due to the recent increase in interest in infection-related topics, following the coronavirus disease 2020 pandemic.

More than half the surgeons (53%) indicated that they would attend these courses if they were part of a major event or conference. Two-thirds (65%) of the participants preferred face-to-face courses compared to online ones. This might be because of the practical nature of these courses, and the overwhelming number of online courses offered during the pandemic. There is also a higher chance of attaining proficiency in these skills with practice, reflection, feedback, and repetition offered in the physical class space [12]. Although face-to-face courses require a greater trainer-participant ratio, it enhances participant-trainer and participant-participant interaction, which can yield better results.

Green et al. highlighted fatigue, stress, and ineffective leadership as the factors that result in human errors in aviation, and this can also be applied to healthcare. They also suggested that ineffective teamwork, communication, and leadership can be added to the list of causes of human errors. One high-risk area is the operating room, as errors here can cause more harm than elsewhere in the healthcare system [31]. Orthopedics is a high-risk specialty, in which errors can occur during the entire management process [32]. A significant number of these errors result from non-technical skill failures in the operating theater, requiring changes in doctors’ practices to enhance patient safety and care [31, 33].

Moreover, several studies have found that poor skills in communication and professionalism, and a lack of teamwork, are significant contributors to adverse events [34, 35]. Addressing this requires a change in culture and current training methods for all operating team members, including surgeons, anesthetists, nurses, and other support staff [36]. This training should be offered with a holistic view of the patient’s needs, and not a disease-focused perspective, to cover multiple aspects of non-technical skills, such as effective communication, professionalism, and team management [6, 31, 37, 38]. Additionally, clinical leadership and managerial skills are reflected in the relevant evidence base, where appropriate leadership features, among other essential non-technical skills, are considered a powerful instrument for improvement [25, 38, 39]. Green et al. considered ineffective leadership to be the third most important cause of errors, after ineffective teamwork and communication [31]. Although leadership was the seventh most important topic in our cohort, it was the fourth most likely skill to receive attendance. Our participants did not see leadership as a critical topic, which could be owing to a lack of understanding of the true meaning of leadership and the difference between managers and leaders [39], or simply due to the need acquire more skills in the other topics that they chose.

### Limitations

The major limitation of this study is that it relies on the self-reported responses of surgeons, which are subjective, and might not reflect the accurate skills of the surgeons as applied in real life. Another limitation is that the tested items might not be comprehensive enough to cover all dimensions of the demands of the non-technical skills. A more objective evaluation will be conducted in phase 2 of this research, including a focus group discussion. However, we can consider the results of this study, which represents a sizable number of orthopedic surgeons, as a basis for future research. Additionally, since the questionnaire enrolled orthopedic surgeons in the MENA region, the results may not be generalizable to orthopedic surgeons in other parts of the world. We acknowledge that patient safety, infection prevention, and control are not skills but patient care outcomes. However, we consider that improving patient safety and infection prevention and control depend on teaching a basic set of skills, such as situation awareness, communication, decision making, and professionalism, which are essential elements of non-technical skills.

## Conclusions

There is a need for non-technical skills training for orthopedic surgeons in the MENA region. Most surgeons prefer these courses to be face-to-face and part of a major event. The most important courses were related to patient safety, infection prevention and control, communication skills, teamwork, and team management.

## Electronic supplementary material

Below is the link to the electronic supplementary material.

Supplementary Material 1

## Data Availability

The datasets used and/or analysed during the current study are available from the corresponding author on reasonable request.
