# Supplementary material for: The need for non-technical skills education in orthopedic surgery
Source: BMC Med Educ. 2023 Apr 19;23:262. doi: 10.1186/s12909-023-04196-2 (PMC10113970; doi:10.1186/s12909-023-04196-2)
Supplement: Supplementary file 1 — Supplementary Material 1 [file 12909_2023_4196_MOESM1_ESM.pdf]

Appendix 1 : Survey questions (electronic survey questions)

Introduction:

Dear colleague

This survey is designed to assess the needs of orthopedic surgeons for Soft Skills Training in the Middle East. The main purpose is to assess the area of interest in this field and ask for your input and suggestions in this type of training.

You will need approximately 4 minutes to finish the survey, and the results of this survey might be published anonymously. By clicking next and starting the survey, we will consider this as consent to participate in this research

Please click the link below to start the survey

Survey questions:

1- What is your major specialty (please select one)?

	Item	Response
1	Orthopedic Trauma	◯
2	Arthroplasty and adult reconstruction	◯
3	Sport medicine	◯
4	Spine surgery	◯
5	Hand surgery	◯
6	Pediatric Orthopedic Surgery	◯
7	Upper Extremity surgery	◯
8	General orthopedic surgery	◯
8	Ilizarov / deformity correction	◯
10	Other (Kindly indicate)	◯

2- Please select your gender:

	Item	Response
1	Female	◯
2	Male	◯

3- Please indicate your age:

20-30 year	31-40 year	41-50 year	51- 60 year	More than 60 year
◯	◯	◯	◯	◯

4- Your working country

	Item	Response
1	Saudi Arabia	◯
2	UAE	◯
3	Egypt	◯
4	Jordan	◯
5	Oman	◯
6	Bahrain	◯
7	Kuwait	◯
8	Tunisia	◯
	Morocco	◯
	Pakistan	◯
	Iran	◯
9	Other	◯

5- Are you board certified in orthopedic surgery?

Yes	No
◯	◯

6- How many years have you been practicing orthopedic surgery?

Less than 5 years	6 to 10 years	11 to 15 years	More than 15 years
◯	◯	◯	◯

7- Do you hold a managerial/ leadership or administrative position in your current working place?

Yes	No
◯	◯

8- What organization type best match your current workplace?

**Governmental hospital**	◯
**Private hospital**	◯
**Academic (University hospital)**	◯
**Health care organization**	◯

9- For each of the following topics, please indicate its importance for you in your clinical practice. (5 very important, 1 useless).

	Item	Very important 5	Important 4	Not- sure 3	Not important 2	Useless 1
1	Medico-legal Issues	◯	◯	◯	◯	◯
2	Communication Skills	◯	◯	◯	◯	◯
3	Clinical Leadership Skills	◯	◯	◯	◯	◯
4	Professionalism	◯	◯	◯	◯	◯
5	Infection Prevention and Control	◯	◯	◯	◯	◯
6	Patient-Doctor Shared Decision Making	◯	◯	◯	◯	◯
7	Conflict Management	◯	◯	◯	◯	◯
8	Patient Safety	◯	◯	◯	◯	◯

9	Teamwork and team management	◯	◯	◯	◯	◯
10	Conflict of Interest	◯	◯	◯	◯	◯
11	Breaking Bad News	◯	◯	◯	◯	◯
12	Doctor-Patient Relationship	◯	◯	◯	◯	◯
13	Telehealth and Virtual patients care.	◯	◯	◯	◯	◯

10- How likely would you attend one-day courses if the course covers one or more of the topics mentioned above?

Very likely (5)	Likely (4)	Not- sure (3)	Not likely(2)	Very unlikely (1)
◯	◯	◯	◯	◯

11- Would you prefer to attend it as independent course or part of major orthopedic event?

independent course	◯
part of major orthopedic scientific activities	◯

1- What is your preferred way to participate in this type of courses?

	Item	Response
1	Face to face activity type of course	◯
2	Online – Virtual Training – course	◯

2- Please indicate how likely you would attend a one-day course in each of the following topics (5 very likely, 1 very unlikely).

	Item	Very likely 5	likely 4	Not- sure 3	Not likely 2	Very unlikely 1
1	Medico-legal Issues	◯	◯	◯	◯	◯

2	Communication Skills	◯	◯	◯	◯	◯
3	Clinical Leadership Skills	◯	◯	◯	◯	◯
4	Professionalism	◯	◯	◯	◯	◯
5	Infection Prevention and Control	◯	◯	◯	◯	◯
6	Patient-Doctor Shared Decision Making	◯	◯	◯	◯	◯
7	Conflict Management	◯	◯	◯	◯	◯
8	Patient Safety	◯	◯	◯	◯	◯
9	Teamwork and Team Management	◯	◯	◯	◯	◯
10	Conflict of Interest	◯	◯	◯	◯	◯
11	Breaking Bad News	◯	◯	◯	◯	◯
12	Doctor-Patient Relationship	◯	◯	◯	◯	◯
13	Telehealth and Virtual patients care.	◯	◯	◯	◯	◯
